# The antileukemic activity of decitabine upon *PML/RARA*-negative AML blasts is supported by all-*trans* retinoic acid: in vitro and in vivo evidence for cooperation

**DOI:** 10.1038/s41408-022-00715-4

**Published:** 2022-08-22

**Authors:** Ruth Meier, Gabriele Greve, Dennis Zimmer, Helena Bresser, Bettina Berberich, Ralitsa Langova, Julia Stomper, Anne Rubarth, Lars Feuerbach, Daniel B. Lipka, Joschka Hey, Björn Grüning, Benedikt Brors, Justus Duyster, Christoph Plass, Heiko Becker, Michael Lübbert

**Affiliations:** 1 Department of Medicine I (Hematology, Oncology and Stem Cell Transplantation), Medical Center, Faculty of Medicine, University of Freiburg, Freiburg, Germany; 2 Institute of Genetic Epidemiology, Medical Center, Faculty of Medicine, University of Freiburg, Freiburg, Germany; 3 Division of Applied Bioinformatics, German Cancer Research Center (DKFZ), Heidelberg, Germany; 4 Faculty of Bioscience, University of Heidelberg, Heidelberg, Germany; 5 Section Translational Cancer Epigenomics, Division of Translational Medical Oncology, German Cancer Research Center (DKFZ) & National Center for Tumor Diseases (NCT), Heidelberg, Germany; 6 Faculty of Medicine, Otto-von-Guericke-University, Magdeburg, Germany; 7 German Cancer Consortium (DKTK), Core Center Heidelberg, Heidelberg, Germany; 8 Division of Cancer Epigenomics, German Cancer Research Center (DKFZ), Heidelberg, Germany; 9 Bioinformatics Group, Department of Computer Science, University of Freiburg, Freiburg, Germany; 10 National Center for Tumor Diseases (NCT), Heidelberg, Germany; 11 German Cancer Consortium (DKTK) and German Cancer Research Center (DKFZ), Partner Site Freiburg, Freiburg, Germany

**Keywords:** Acute myeloid leukaemia, Preclinical research, Cancer models

## Abstract

The prognosis of AML patients with adverse genetics, such as a complex, monosomal karyotype and *TP53* lesions, is still dismal even with standard chemotherapy. DNA-hypomethylating agent monotherapy induces an encouraging response rate in these patients. When combined with decitabine (DAC), all-*trans* retinoic acid (ATRA) resulted in an improved response rate and longer overall survival in a randomized phase II trial (DECIDER; NCT00867672). The molecular mechanisms governing this in vivo synergism are unclear. We now demonstrate cooperative antileukemic effects of DAC and ATRA on AML cell lines U937 and MOLM-13. By RNA-sequencing, derepression of >1200 commonly regulated transcripts following the dual treatment was observed. Overall chromatin accessibility (interrogated by ATAC-seq) and, in particular, at motifs of retinoic acid response elements were affected by both single-agent DAC and ATRA, and enhanced by the dual treatment. Cooperativity regarding transcriptional induction and chromatin remodeling was demonstrated by interrogating the *HIC1*, *CYP26A1*, *GBP4*, and *LYZ* genes, in vivo gene derepression by expression studies on peripheral blood blasts from AML patients receiving DAC + ATRA. The two drugs also cooperated in derepression of transposable elements, more effectively in U937 (mutated *TP53*) than MOLM-13 (intact *TP53*), resulting in a “viral mimicry” response. In conclusion, we demonstrate that in vitro and in vivo, the antileukemic and gene-derepressive epigenetic activity of DAC is enhanced by ATRA.

## Introduction

DNA-hypomethylating agents (HMAs) are well-established treatment options for patients with AML and MDS, also in the presence of adverse genetics. However, as single-agent HMA treatment is slow-acting, with a prolonged time to best response, and an overall modest response rate, two-drug combinations have been clinically advanced over the last years. Cooperative activities (recently reviewed by Stomper and colleagues [1]) include BCL-2 inhibition (by ABT-199/venetoclax) and retinoid signaling (with all-*trans* retinoic acid (ATRA) or RARA agonist tamibarotene), among others. Retinoids such as ATRA are attractive combination partners also by virtue of their favorable toxicity profile, allowing treatment also of elderly and frail patients. Sensitization of non-APL AML to retinoids (as recently reviewed by Geoffroy and colleagues [2]) was the goal of several non-randomized clinical trials combining ATRA with azacitidine (AZA) [3, 4] or decitabine (DAC) [5] and tamibarotene with AZA [6]. In a randomized phase II study (DECIDER trial) [7] we demonstrated a significantly increased response rate and improved median overall survival (from 5.1 to 8.2 months) in patients receiving ATRA added to DAC; of note, this benefit was observed not only in patients with non-adverse but also in those with adverse genetics.

Therefore, in the present study we tested the hypothesis that ATRA cooperates with DAC, resulting in initiation of transcriptional programs triggering antileukemic activity, including reactivation of transposable elements (TEs), thus unleashing a “viral mimicry” antitumoral response. To model our clinical results (obtained in *PML-RARA*-negative AML patients), we employed the U937 cell line model representing *TP53*-mutated AML (constituting a substantial proportion of elderly AML patients) and MOLM-13 representing AML with functional p53. We demonstrate at least additive activity of DAC and ATRA on the cellular and transcriptional level, with increased chromatin accessibility, and massive reactivation of TEs, particularly in *TP53*-mutated U937 cells. Cooperative effects between the HMA and the retinoid could also be confirmed in primary AML blasts, serially isolated from peripheral blood of AML patients receiving this drug combination. The study thus supports further preclinical and clinical investigation of HMA and retinoid combination therapies.

## Materials and methods

### Cell culture and drug treatment

Cell lines (U937, MOLM-13, OCI-AML3, MV4-11, THP1, HL-60) were treated with three daily pulses of 5-aza-2′-deoxycytidine (decitabine, DAC, Selleckchem, Houston, U.S.A.) dissolved in PBS. 1 µM all*-trans* retinoic acid (ATRA, Sigma-Aldrich, St. Louis, U.S.A.), dissolved in DMSO, was administered on day 3 simultaneously with the third dose of Decitabine (48 h after the first dose of DAC). For details see Supplemental Methods.

### Caspase-3/7 activity assay

See Supplemental Methods.

### RNA-sequencing and data analysis

Cells were treated with DAC and ATRA and harvested at 72 h. Total RNA was isolated as previously described [8] and depleted of ribosomal RNA (Ribo-Zero Gold rRNA Removal Kit, Illumina). cDNA libraries (strand-specific, 50 bp) were sequenced on an Illumina HiSeq2500 sequencer with 35 million paired-end reads per sample. For data analysis see Supplemental Methods.

### Assay for transposase-accessible chromatin (ATAC)-sequencing and data analysis

For ATAC-sequencing (ATAC-seq), 50,000 fresh cells were processed in triplicates as described in the Omni-ATAC protocol by Corces and colleagues [9]. For details and data analysis see Supplemental Methods.

### DNA methylation profiling by Infinium HumanMethylation450 BeadChip Array

Methylomes were generated using Infinium Human Methylation 450 K BeadChip arrays. Background subtraction was performed using the methylumi package (method “enmix.oob”) [10] and beta-mixture quantile normalization (BMIQ) [11]. Differential methylation data was obtained as previously described [8]. For details see Supplemental Methods.

### LINE-1 methylation

LINE-1 methylation was quantified at three selected CpGs by MassArray, as previously described by Claus and colleagues [12].

### AML patient blasts and expression analyses

Peripheral blood mononuclear cells (PBMC) were collected from 15 AML patients; 9 treated with DAC only and 6 treated with DAC + ATRA within the DECIDER trial [7] (NCT00867672). Leukemic blood blasts before treatment and at day 8 were isolated using automatic magnetic sorting of cells labeled with anti-human anti-CD34 and CD117 MACS microbeads. RNA isolation, qRT-PCR and expression profiling with Human Gene 2.0 arrays (Affymetrix) were performed as previously described [8]. Patients provided written informed consent for the research use of the clinical data and biomaterial in accordance with the Declaration of Helsinki. Expression array data are available at GEO under accession number GSE171053.

### Quantitative qRT-PCR

See Supplemental Methods.

### Fluorescent western blot

See Supplemental Methods.

### Fetal hemoglobin quantification from erythrocytes of patients receiving decitabine-based treatment by high-performance liquid chromatography

HbF levels from patients treated on the DECIDER trial were measured, as previously described [13], by high-performance liquid chromatography (HPLC), and the following time points were analyzed: before treatment and after the end of the second treatment course.

### Raw data access

RNA-sequencing, chromatin accessibility profiling and expression array data are available at GEO under accession numbers GSE184324, GSE184282, GSE190878 and GSE171053, respectively. Methylome data (arrays) are available under accession number GSE181960.

### Statistical analyses

Statistical analyses were performed with GraphPad Prism V8.3 (San Diego, California, U.S.A.).

## Results

### Decitabine (DAC) and all-*trans* retinoic acid (ATRA) disclose cooperative antileukemic activity, inducing transcriptome changes in AML cell lines

**Fig. 1:**
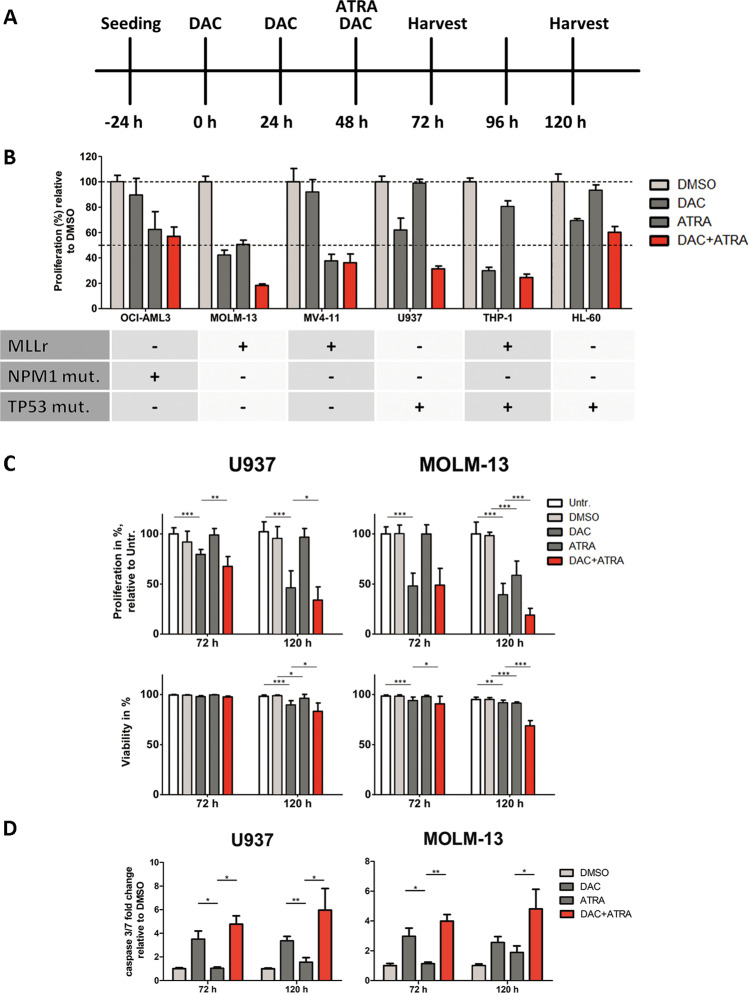
Decitabine and all-*trans* retinoic acid exhibit cooperative effects on inhibition of proliferation and reduction of viability in AML cell lines. **A** Treatment scheme. **B** Baseline-corrected proliferation of OCI-AML3, MOLM-13, MV4-11, U937, THP1, and HL-60 cells treated with DAC (100 nM) and ATRA (1 µM) after 120 h. Experiments were conducted in technical triplicates. **C** Proliferation and viability of U937 and MOLM-13 cells were measured 72 and 120 h after the first dose of Decitabine, untreated or treated with DMSO, DAC (U937: 200 nM; MOLM-13: 100 nM), ATRA (1 µM) or DAC + ATRA in combination. Three independent experiments, each with three independent technical replicates, were conducted. Standard deviation is shown as error bars. **D** Caspase-3/7 activity in U937 (left panel) and MOLM-13 cells (right panel) displayed as fold-change relative to a DMSO-treated control at 72 and 120 h. Six (72 h) and nine (120 h) independent experiments, each with three independent technical replicates, were conducted, respectively. Standard deviation is shown as error bars. Statistical significance was tested for by unpaired *t*-test with a threshold of *p* < 0.05.

Among six AML cell lines (3 with mutated, 3 with wildtype *TP53*) treated with DAC and ATRA, single-agent activity upon cell growth inhibition was variable (with notable ATRA sensitivity of the three cell lines with MLL rearrangements), without antagonistic effects (Fig. 1A, B). U937 and MOLM-13 were selected for further study due to the strongest drug cooperation, with U937 disclosing a *TP53* mutation, MOLM-13 (wildtype *TP53*) a translocation (9;11) with MLL-AF9 fusion. Combining DAC with ATRA resulted in higher time- dependent growth inhibition and stronger reduction in viability (particularly at 120 h) than either drug alone (Fig. 1C). The observed cytotoxicity induced by the dual treatment was indicative of apoptosis, which was confirmed by significant activation of caspases 3 and 7 at 72 and 120 h (Fig. 1D).

**Fig. 2:**
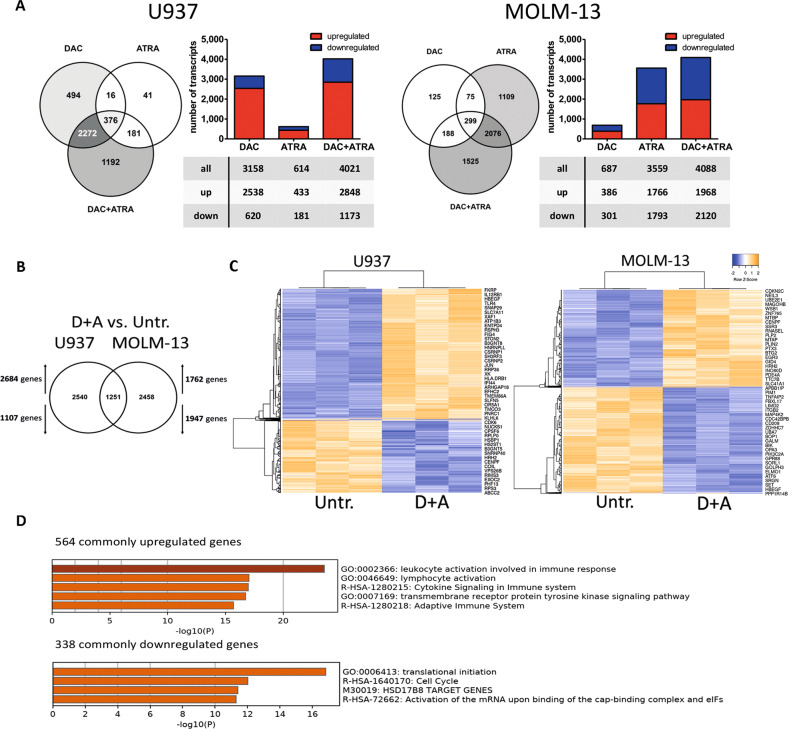
Decitabine and all-*trans* retinoic acid cooperate in inducing transcriptome changes in AML cells. **A** Global expression changes in U937 and MOLM-13 were measured by RNA-seq (FDR < 0.01). Cells were treated with DAC, ATRA or both in combination, and harvested 72 h after the first dose of Decitabine. Bar and Venn diagrams depict the numbers of altered transcripts in comparison to untreated; upregulated transcripts are shown in red, downregulated in blue. **B** Comparison of significantly regulated genes in U937 and MOLM-13 treated with DAC + ATRA vs. untreated (determined by RNA-seq). **C** Heatmaps (unsupervised clustering) of the commonly regulated genes in U937 or MOLM-13, comparing untreated (untr.) to DAC + ATRA-treated cells. **D** GO analysis for the commonly up- or downregulated genes in U937 and MOLM-13 by DAC + ATRA.

To investigate early transcriptome changes induced by these treatments, i.e., before the onset of secondary effects, U937 and MOLM-13 were analyzed by RNA-sequencing at the 72 h time point. U937 revealed DAC sensitivity, with the majority of transcripts being upregulated, whereas the effect of ATRA was limited; the combination resulted in an additive effect. MOLM-13 cells disclosed a limited effect of DAC, but were quite sensitive to ATRA, also with an additive effect of the combination (Fig. 2A). Notably, approximately one-third of the transcripts regulated by DAC + ATRA in each cell line was shared, i.e., 1251 commonly regulated genes (Fig. 2B), with 902/1251 transcripts (72.1%) in the same direction. Co-regulated transcripts are depicted by unsupervised clustering (Fig. 2C). As expected, more genes were induced in U937 compared to MOLM-13 (and vice versa); GO analysis unveiled a marked enrichment for upregulated transcripts involved in leukocyte differentiation and immune response, and downregulated transcripts associated with translational initiation (Fig. 2D).

### Synergistic transcriptional induction of retinoic-acid responsive genes by DAC and ATRA is associated with increased chromatin accessibility

**Fig. 3:**
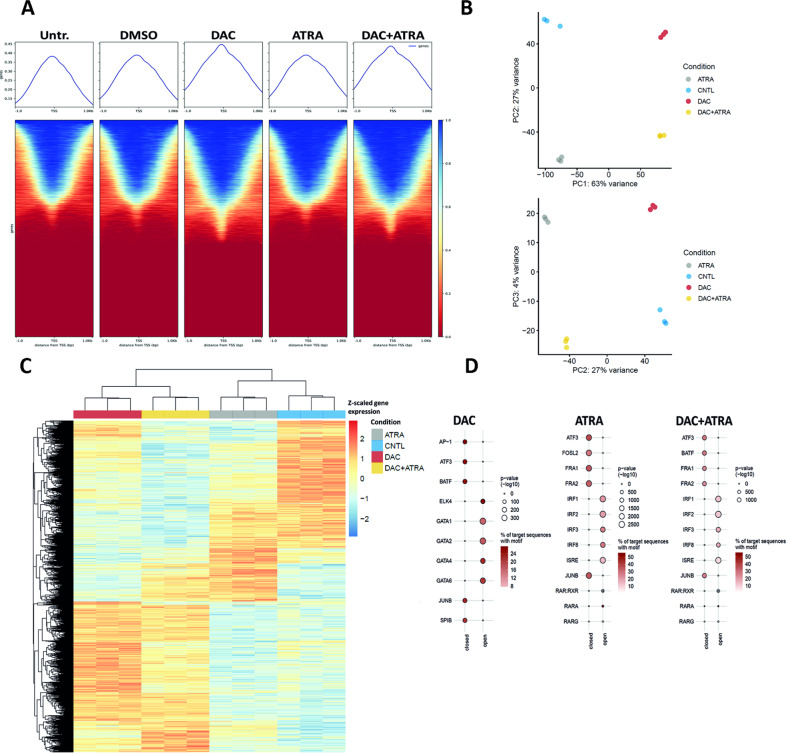
Cooperation between DAC and ATRA in modulating chromatin accessibility. **A** Enrichment of accessible chromatin sites across the whole genome determined by ATAC-sequencing in U937 cells (technical triplicates) either untreated (untr.) or treated with DMSO, DAC, ATRA or DAC + ATRA at 72 h. Sequencing was performed with 40 million reads per sample (paired-ends). Mean alignment was at about 99%, with 51–63% uniquely mapped sequences. **B** Principal component (PC) analysis (PCA) of the assay for transposase-accessible chromatin sequencing (ATAC-seq) data of control (untreated, CNTL; *n* = 3), ATRA (*n* = 3), DAC (*n* = 3), and DAC + ATRA-treated (*n* = 3) samples. Chromatin accessibility of the 200,000 most variably accessible peaks was used for dimensionality reduction. **C** Heatmap of the 50,000 most variable differentially accessible regions (DARs) from the ATRA vs. untreated (CNTL), DAC vs. CNTL, and ATRA + DAC vs. CNTL comparison. Euclidean distance of the z-scaled chromatin accessibility of DARs in all samples was visualized. **D** Homer transcription factor motif enrichment of differentially accessible regions (adj. *p*-value < 0.05 and absolute log 2 fold-change >1), stratified in open and closed DARs. The top five most significantly enriched transcription factor motifs as well as all significantly enriched (adjusted *p*-value < 0.05) retinoic acid response (RAR) motifs are visualized. The union set of all peaks was used as a background for enrichment analyses.

We hypothesized that single-agent DAC, ATRA or the combination of both may alter chromatin accessibility [14], and thus performed ATAC-seq on U937 cells. As shown in Fig. 3A, globally, DAC alone had a much stronger effect on chromatin accessibility than ATRA alone.

However, when focusing on the 200,000 most variably accessible peaks for dimensionality reduction, a principal component analysis (Fig. 3B) revealed a distinct grouping for each treatment, clearly separating DAC- from non-DAC-treated (PC1, explaining 63% of the variability) and ATRA- from not-ATRA-treated samples (PC2; explaining 27% of the variability). These results indicate a global effect of both DAC and ATRA, as single agents, on chromatin accessibility, as confirmed in a differential accessibility analysis (Suppl. Fig. 2). Visualization of the 50,000 most variable differentially accessible regions (Fig. 3C) revealed regions of chromatin opening induced by DAC but not ATRA and, in a smaller population, by ATRA but not DAC; for a subset of peaks, both drugs cooperated in chromatin opening. Regarding chromatin closing, activity was noted not only for DAC, but also for ATRA, and here also, both drugs showed cooperative activity for a substantial number of regions.

Next, we asked whether increased chromatin accessibility either by single-agent treatment with DAC or ATRA, or by combination treatment, was associated with transcription factor (TF) and retinoic acid response (RAR) motifs. Indeed, TF motif analysis (Fig. 3D) revealed a drug-specific enrichment, with chromatin opening by ATRA, but not DAC, for IRF1, -2, -3, -8 and ISRE (middle panel), whereas with DAC, the 5 TF motifs with the highest degree of chromatin opening included GATA1, -2, -4, -6, and ELK4 (left panel). Upon dual treatment, the ATRA signature for chromatin opening remained stable (right panel). Visualizing drug effects on chromatin opening at retinoic acid response elements (RAREs), single-agent ATRA, but not DAC, resulted in chromatin opening at the RAR/RXR, RARA and RARG motifs; here also, the ATRA signature could also be discerned with the combined treatment.

Next, we selected individual genes bearing RAREs to interrogate regions of differential chromatin accessibility. Three genes with known RAREs and CpG-rich regions (*HIC1, CYP26A* and *GBP4*) were selected from the genes disclosing the highest levels of induction by DAC + ATRA. *HIC1* is a well-established tumor suppressor gene [15], with ATRA-responsiveness previously demonstrated in AML [16], *CYP26A1* is central in ATRA metabolism [17], and *GBP4* represents an interferon-response gene recently described as an ATRA-regulated target of IRF1 [18]. In addition, we also interrogated *LYZ*, a DAC-sensitive gene [19] (also with an RARE, but lacking a CpG island) that others as well as ourselves described as epigenetically regulated during myelopoiesis [20], with DAC-induced chromatin opening [21].

**Fig. 4:**
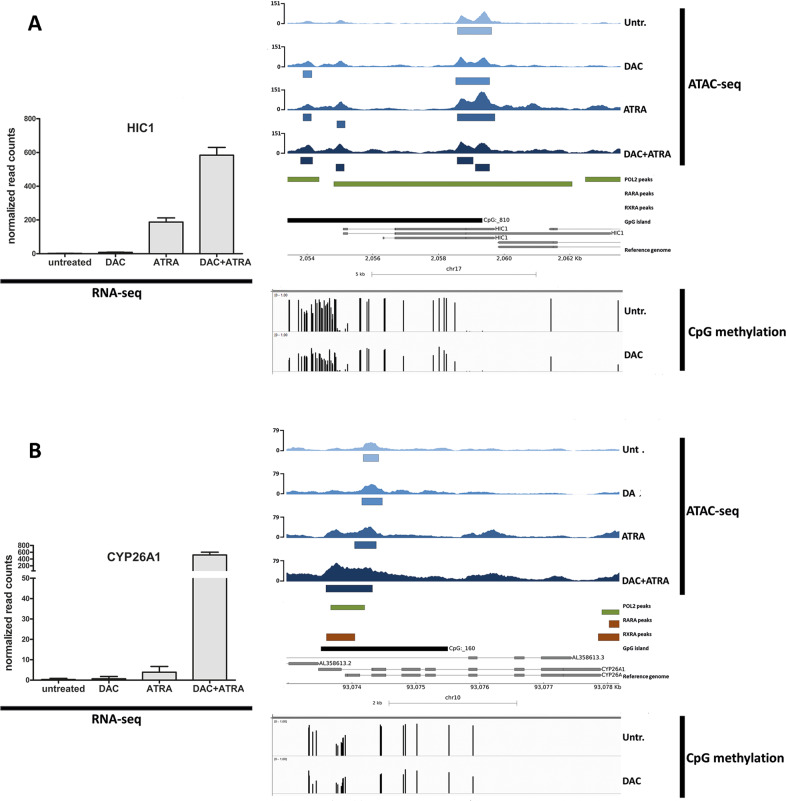
Transcriptional induction of *HIC1* and *CYP26A1* by DAC and ATRA is associated with changes in chromatin accessibility. **A**, **B** mRNA expression (normalized read-counts) of *HIC1* (**A**) and *CYP26A1* (**B**) of both untreated U937 cells and U937 after treatment with either DAC, ATRA, or DAC + ATRA at 72 h was determined by RNA-seq (left panel). Chromatin accessibility of these cells and treatment modes was determined by ATAC-seq (upper right panel). Blue boxes below the ATAC-seq track represent significant effects. Annotations for POLR2A binding sites (green boxes), retinoic acid receptors RARA and RXRA (brown boxes) and CpG islands (black boxes) were extracted from ChiP-seq data accessed from ENCODE and GEO (right panel). CpG methylation data of U937 cells untreated or treated with DAC were determined by Infinium HumanMethylation450 BeadChip Assay [8] (lower right panel).

**Fig. 5:**
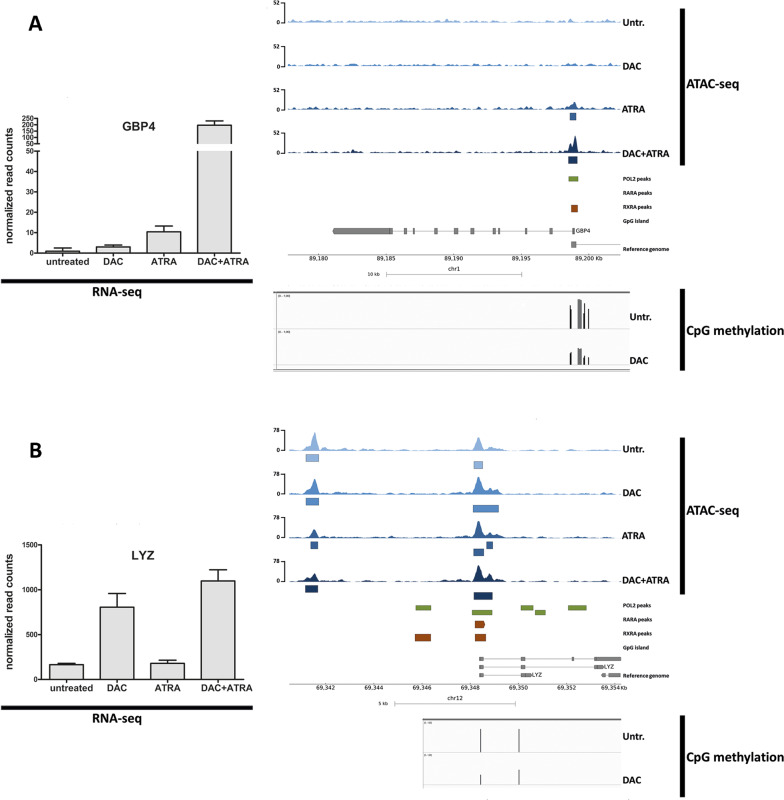
Transcriptional induction of *GBP4* and *LYZ* by DAC and ATRA is associated with changes in chromatin accessibility. **A**, **B** mRNA expression (normalized read-counts) of *GBP4* (**A**) and *LYZ* (**B**) of both untreated U937 cells and U937 after treatment with either DAC, ATRA, or DAC + ATRA at 72 h was determined by RNA-seq (left panel). Chromatin accessibility of these cells and treatment modes was determined by ATAC-seq (upper right panel). Blue boxes below the ATAC-seq track represent significant effects. Annotations for POLR2A binding sites (green boxes), retinoic acid receptors RARA and RXRA (brown boxes) and CpG islands (black boxes) were extracted from ChiP-seq data accessed from ENCODE and GEO (right panel). CpG methylation data of U937 cells untreated or treated with DAC were determined by Infinium HumanMethylation450 BeadChip Assay [8] (lower right panel).

As shown in Figs. 4 and 5, the dual treatment resulted in synergistic transcriptional upregulation of all four genes, with increased chromatin accessibility compared to untreated cells. Single-agent treatment with either DAC or ATRA resulted in variable induction as well as chromatin opening. This is exemplified by a 5-fold induction of *LYZ* mRNA by DAC, with concomitant broadening of the ATAC-seq peak around *LYZ* exon 1 (Fig. 5B). Across all four genes, the seven peaks representing significantly treatment-induced higher chromatin accessibility are marked by blue boxes beneat the ATAC-seq tracks; the majority of regions with treatment-induced increased chromatin accessibility co-localized with Pol2 peaks, RARA, RXRA and CpG islands. This is in line with ATRA acting within a transcriptional activator complex, directly involved in initiation of transcription, in these genes selected for known retinoic acid responsiveness.

Three of these four genes have CpG islands of variable size, with *HIC1* disclosing the by far largest island (composed of 810 CpGs, compared to 160 CpGs in the *CYP26A1* promoter and 5’ coding region), *GBP4* also exhibiting a CpG-dense promoter region (albeit not fulfilling CpG island criteria), *LYZ* lacking a bona fide CpG island [21]. We asked whether U937 cells treated with DAC disclose DNA hypomethylation at these regions, analyzing Illumina array data [8]. Indeed, demethylation was observed for all four genes (Figs. 4 and 5, lower panels), co-localizing with regions of increased chromatin accessibility.

### Global DNA demethylation by decitabine is not enhanced by all-*trans* retinoic acid

**Fig. 6:**
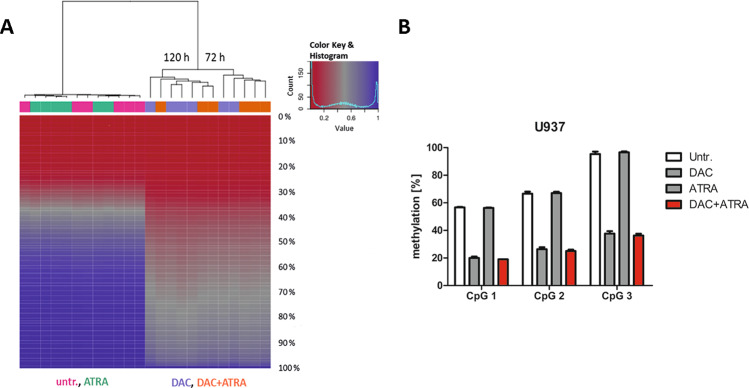
Global DNA demethylation by DAC is not enhanced by ATRA. **A** U937 cells (technical triplicates) were treated with either DAC (lilac), ATRA (green), DAC + ATRA (orange) or left untreated (untr.; pink) and analyzed after 72 and 120 h. Global DNA methylation was acquired by Infinium HumanMethylation450 BeadChip Assay [8]. Methylation data was analyzed with RnBeads as described in Materials and Methods and Suppl. Methods. The heatmap displays the methylation percentiles (%) for all 24 samples analyzed. Unsupervised, hierarchical clustering showed subclustering in the DAC/DAC + ATRA arm for the two time points with an outlier in the 120 h group. **B** LINE-1 methylation of three selected CpGs was determined by MassArray in U937 cells (technical triplicates) treated with DAC, ATRA or DAC + ATRA at 72 h.

Given evidence in the literature that ATRA may also act by inhibiting DNA methyltransferase activity, we wished to pursue this hypothesis using a methylome-wide approach. Methylation in U937 was analyzed by Infinium HumanMethylation450 BeadChip Array [22] at 72 und 120 h (Fig. 6A). The massive demethylating effect in gene bodies and promoters of the DNMT inhibitor DAC could be detected both for the single-agent treatment as well as when combined with ATRA. Correlation-based clustering revealed a subclustering, in the DAC/DAC + ATRA arm, for time point of harvest (72 and 120 h) with one outlier (DAC sample harvested at 72 h) in the 120 h group.

Global DNA methylation can also be detected by the methylation status of transposable repetitive LINE elements. These make up 21% of the human genome and are highly methylated. These properties make them an ideal surrogate for genome-wide methylation status. Focusing on three informative CpGs within the LINE-1 promoter, we quantified methylation by MassArray [23]. At 72 h, untreated and ATRA-treated cells showed a comparable degree of methylation, whereas DAC-only- and DAC + ATRA-treated cells showed similar, marked demethylation across all three CpGs compared to untreated cells (Fig. 6B). Thus, we conclude that also by this approach, no activity of ATRA in demethylating DNA could be discerned.

### Gene induction in AML peripheral blood blasts during treatment with DAC and ATRA

Observing cooperative, in part synergistic effects of DAC and ATRA on gene transcript expression, we considered that this may be observed, at least to some degree, also in vivo. Peripheral blood blasts from nine AML patients treated with DAC alone for 5 days, and six patients treated with DAC followed by ATRA initiated on day 6 (DECIDER trial [24]) were interrogated by cDNA array analyses. *TP53* was mutated in three patients treated with DAC alone and none of the patients receiving DAC + ATRA (Suppl. Table 3). Therefore, comparability of *TP53* mutation status was limited for this cohort.

**Fig. 7:**
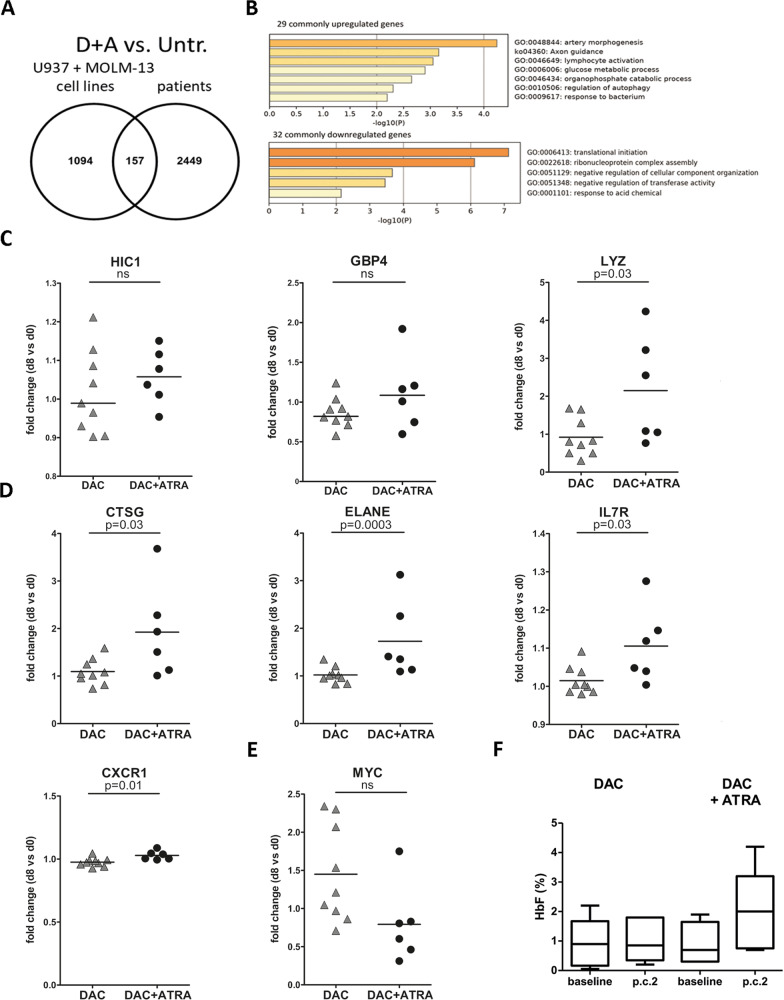
Changes in primary blasts of AML patients treated with decitabine and all-*trans* retinoic acid. **A** Venn-diagram of transcriptionally regulated genes in U937 and MOLM-13 cells (determined by RNA-seq) and AML patients (*n* = 6) treated with DAC + ATRA (primary AML blasts; expression arrays performed on day 0 and day 8, compared to untreated). **B** GO analysis of up- or downregulated genes, in U937 and MOLM-13, and primary AML blasts. **C** Scatter plots of *HIC1*, *GBP4* and *LYZ* expression changes in patient samples (day 8 vs. day 0) having received DAC only (*n* = 9) or DAC + ATRA (*n* = 6). Fold-changes are displayed. Statistical significance was tested for by unpaired t-test with a threshold of *p* < 0,05 (ns, not significant). **D** Scatter plots displaying expression changes in *CTSG*, *ELANE, IL7R* and *CXCR1* as described in Fig. 7C. **E** Scatter plot of *MYC* expression changes (downregulated by DAC + ATRA compared to DAC alone) as described in Fig. 7C. **F** Levels of fetal hemoglobin (HbF,%) in AML patients’ peripheral blood erythrocytes before treatment (baseline) and after 2 courses (p.c.2) of treatment with either DAC only (*n* = 6) or DAC + ATRA (*n* = 5) were determined by HPLC as described in Materials and Methods.

The two time points compared for changes in transcript expression were: i. prior to and ii. at day +8 from treatment start (48 h after initiation of ATRA). As shown in Fig. 7A, 157 genes regulated by DAC + ATRA were shared by all six patients and the two AML cell lines (see Fig. 7B for GO analyses). For *HIC1*, *GBP4* and *LYZ*, median mRNA expression changes at day 8 tended to be higher in patients receiving the dual treatment compared to those receiving single-agent DAC (Fig. 7C) with a statistically significant difference reached for *LYZ*, whereas no difference was observed for *CYP26A1* (not shown).

As shown in Fig. 7D, significantly higher fold-changes (FCs) with DAC + ATRA treatment compared to DAC alone were also noted for several other genes with important roles during myeloid differentiation: cathepsin G (*CTSG;* mean FC DAC + ATRA 1.92 vs. mean FC DAC 1.09; Suppl. Table 4), neutrophil elastase (*ELANE*; mean FC DAC + ATRA 1.73 vs. mean FC DAC 1.02; Suppl. Table 4), the IL7 receptor (*IL7R;* mean FC DAC + ATRA 1.11 vs. mean FC DAC 1.02; Suppl. Table 4) and the interleukin-8 receptor *CXCR1 (CXCR1;* mean FC DAC + ATRA 1.03 vs. mean FC DAC 0.97). On the other hand, numerous genes were downregulated by DAC + ATRA (Suppl. Table 5), including *MYC* (mean FC DAC + ATRA 0.79 vs. mean FC DAC 1.45; Fig. 7E and Suppl. Table 5).

### In vivo induction of fetal hemoglobin by continued treatment with DAC and ATRA

Since these patients had received only limited (48 h) ATRA exposure, we asked whether upregulated fetal hemoglobin (HbF), one of the few biomarkers for HMA response [25], would undergo more pronounced kinetics of gene induction than observed after 48 h of ATRA. HbF was serially measured before treatment start and after 2 treatment courses (i.e., after 46 days of ATRA intake). As shown in Fig. 7F, the median HbF protein levels measured in patients’ peripheral blood erythrocytes were not altered for six patients not receiving ATRA as an add-on to DAC (0.9 vs. 0.9%) and rose from 0.7 to 2.0% in five patients receiving DAC + ATRA (paired *t*-test *p* = 0.0632, not significant).

### Decitabine and all-*trans* retinoic acid cooperate in derepressing transposable elements in AML cells, activating dsRNA sensing

**Fig. 8:**
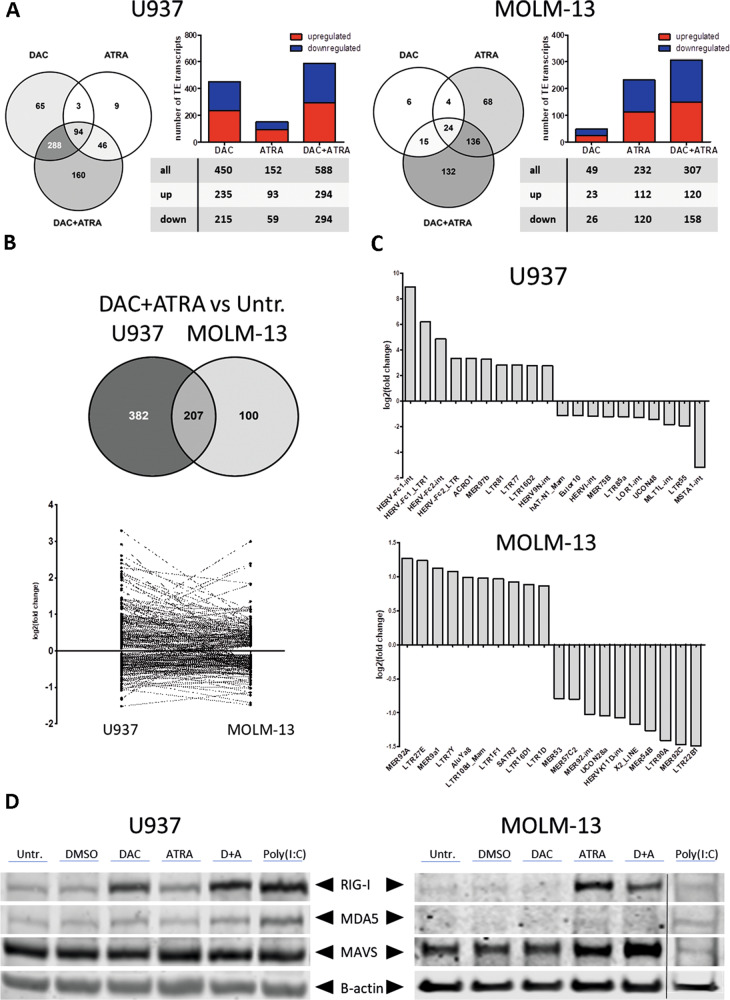
Decitabine and ATRA cooperate in derepressing transposable elements in AML cells, activating dsRNA sensing. **A** Global expression changes of TEs in U937 and MOLM-13 were measured by RNA-seq (FDR < 0.05). Cells were treated with DAC, ATRA or both in combination and harvested 72 h after first dose of Decitabine. Bar and Venn diagrams show the numbers of altered TEs for each treatment (DAC, ATRA, DAC + ATRA) in comparison to untreated; upregulated transcripts shown in red, downregulated in blue. **B** Comparison of regulated TEs in U937 and MOLM-13 treated with DAC + ATRA vs. untreated determined by RNA-seq. 102 out of 207 TE (49.3%) transcripts show concordance regarding induction vs. repression, as depicted in the lower graph. **C** Waterfall plots of the 10 most up- or downregulated TEs in U937 or MOLM-13 treated with DAC + ATRA. **D** Immunoblot results for RIG-I, MDA5 and MAVS in whole-cell lysates of U937 and MOLM-13 treated as indicated above. Beta-actin was used as loading control; the space between protein bands was cropped to conserve space. Results for the poly(I:C) sample in MOLM-13, repeated due to technical issues, were replaced.

The ability of HMAs to reactivate transposable elements (TEs) in cancer cells, thereby triggering antitumoral responses (“viral mimicry”), has been demonstrated in several recent publications [24, 26–28]. Given that repetitive elements may also possess RAREs, we hypothesized that ATRA (alone and in combination with DAC) may also reactivate TE expression [29]. Analysis of U937 and MOLM-13 for TE expression [24] revealed that the total number of differentially expressed TEs (Fig. 8A) mirrored the global effects on transcription shown in Fig. 2A, with U937 disclosing predominant sensitivity to single-agent DAC but not ATRA, MOLM-13 with predominant sensitivity to single-agent ATRA but not DAC. Combining both drugs resulted in synergistic effects in both cell lines, with a balanced ratio of up- vs. downregulated TEs. For U937, TEs also regulated in MOLM-13 amounted to approximately one-third of all sequences, and two-thirds of TEs regulated in MOLM-13 overlapped with those regulated in U937 with concordant up- or downregulation for most of them (Fig. 8B). In U937, the 10 most strongly induced TEs surpassed the 10 most strongly repressed TEs by amplitude, whereas in MOLM-13 cells, the fold-changes (overall lower than in U937) of induction vs. repression were in a similar range (Fig. 8C).

As we have recently demonstrated co-regulated induction of the endogenous retrovirus *ERV3-1* and the zinc finger protein *ZNF117* in different AML cell lines including U937 by DAC [24], it was of interest to interrogate these two genes also for the effect of ATRA. We observed that *ERV3-1* RNA was induced about threefold at two different time points by ATRA alone, and expression was further induced by the DAC + ATRA combination treatment. As expected, similar kinetics of induction were noted for *ZNF117* (Suppl. Fig. 3A).

Since dsRNAs induced by epigenetic treatment trigger an antiviral immune response via binding to RIG-I or MDA5 and the complex then binding to the dsRNA sensor MAVS at the mitochondrium (Suppl. Fig. 3B), we investigated expression and interaction of these three components of the antiviral cellular response machinery. In U937, RIG-I mRNA (data not shown) and protein were readily induced by DAC and modestly by ATRA (Fig. 8D, left panel, poly(I:C) was used as a positive control). The drug combination led to further induction. In MOLM-13 cells, ATRA was much more effective than DAC in inducing RIG-I protein (Fig. 8D, right panel). Regarding MDA5 protein levels, induction was modest for both cell lines. MAVS protein was abundant in untreated cells; levels were unaffected in U937 cells but were clearly induced in MOLM-13 (ATRA, DAC + ATRA). The protein-protein interaction, interrogated by co-immunoprecipitation, remained stable under the different treatment conditions (not shown). Thus, we conclude that a “viral mimicry” response, a recently uncovered antineoplastic mechanism triggered by HMAs, can be enhanced by ATRA (possibly dependent on the *TP53* status of the leukemic cells) [30, 31].

## Discussion

In elderly, medically non-fit AML/MDS patients, the exploration of combination therapies with an HMA backbone is aimed at low- to non-toxic combination partners given the age-related clinical limitations of these patients. One rational approach to identify effective dual treatment is an exploitation of the “priming” capacity of HMAs, by inducing DNA hypomethylation and thus derepressing genes involved in pathways that are aberrantly silenced by hypermethylation in the malignant cells. Given the ability of ATRA to induce senescence, differentiation and apoptosis in AML cell lines [32–35] and to control dormancy vs. differentiation in normal hematopoietic stem cells (HSCs) [36, 37], we have conducted a randomized clinical trial, demonstrating a clinically meaningful survival benefit in elderly, non-fit AML patients treated with DAC and ATRA compared to DAC treatment without ATRA [7]. Encouraging clinical experience was also gained in several single-arm studies combining DAC or AZA with ATRA [3] or with the RARA-agonist tamibarotene [38]. It was therefore of great interest to arrive at a better understanding of the underlying mechanisms of action for this clinically significant in vivo interaction between HMAs and retinoids, which may potentially be relevant also in other malignancies.

We demonstrate cooperativity between both drugs in two AML cell lines, one—U937—with mutated *TP53*, the other—MOLM-13—with wildtype *TP53*, regarding inhibition of proliferation, induction of cell death, and coordinate regulation of more than 1200 transcripts (the majority becoming derepressed). As expected, single-agent DAC induced chromatin accessibility in ~31,000 regions. ATRA also displayed this activity, albeit at distinct regions (frequently containing RAREs), and dual treatment resulted in cooperative effects on chromatin opening in a subset of regions. Somewhat surprisingly, cooperativity between both agents was also noted for chromatin closing in ~30,000 regions. Further investigations appear warranted to characterize the mechanisms underlying the effects of ATRA upon chromatin structure, since epigenetic activities of retinoids are as yet understudied.

In this regard, for a second functional layer of antileukemic activity being mediated by ATRA, it may be hypothesized that the retinoid might support the DNMT-inhibitory activity of DAC, since several studies have described at least partial demethylation of specific genes with ATRA alone [39, 40]. However, when we addressed this question at the methylome level, no global demethylation was detected upon single-agent ATRA, nor did ATRA enhance the demethylation mediated by DAC. However, since we used methylation arrays, we cannot exclude that a subpopulation of genes is more effectively demethylated by the combination of DAC + ATRA compared to DAC alone. Specifically, we cannot rule out that in regulatory regions of genes undergoing chromatin opening and transcriptional activation in response to ATRA, demethylation of hypermethylated CpG islands may occur.

Striking cooperativity on transcriptional derepression in U937 cells was observed for the tumor suppressor gene *HIC1*, a well-established ATRA target [15], and (as its acronym indicates), hypermethylated in many types of cancer, particularly in those with inactivation of a copy of p53 [41]. We could previously show [24] that in *TP53-*mutated ELF-153 AML cells that have lost one allele of 17p (i.e., the region bearing both the *TP53* and *HIC1* loci), *HIC1* expression from the remaining allele was particularly strong upon DAC treatment. *HIC1* reactivation in vitro and in vivo (in serially sorted primary AML blood blasts from DAC-treated patients) was more pronounced in haploinsufficient AML cells compared to cells with both *HIC1* alleles present. This observation argues for a particular sensitivity of this tumor suppressor gene locus to DAC-induced derepression when in a monoallelic state [24]. It is tempting to speculate that the >500 fold induction of *HIC1* mRNA in U937 cells treated with the DAC + ATRA combination may mediate, at least in part, its antileukemic effect; inactivation and overexpression studies of *HIC1* would be necessary to test this hypothesis.

When pursuing in vivo validation of results generated in vitro, it has to be considered that compared to the often striking mRNA expression changes determined under optimized conditions of cell culture, in vivo effects of epigenetic drugs determined in primary cells from patients undergoing epigenetic therapy tend to be more modest; reasons are manifold, including variable serum drug levels, variable drug uptake etc [24]. Despite these limitations and the drug exposure to ATRA being limited at time of analysis (analysis on day 8; 2 days after first ATRA intake on day 6) we observed upregulation of several genes with DAC plus ATRA (vs. DAC alone), not only *HIC1* and CYP26A1, but also *LYZ*, *CTGS* and other myelopoiesis-associated genes regulated during differentiation. Within the DECIDER trial, in vivo differentiation was not systematically studied by serial NGS analyses; however, monitoring clone size reduction by FISH, we could replicate previous results [42] demonstrating hematologic improvement in the presence—i.e., delayed clearance—of the clonal marker, in line with in vivo differentiation, in several patients treated with DAC and ATRA (data not shown).

To also interrogate later time points after longer intake of ATRA, we evaluated kinetics of HbF induction, demonstrating stronger HbF increments in DAC + ATRA-treated patients compared to those receiving DAC alone, after 2 treatment courses (i.e., 7 weeks from ATRA treatment start). Thus, serial determination of HbF as a dynamic biomarker may aid in response monitoring during HMA treatment when combined with a second gene-reactivating agent.

The in vivo mechanism of action of HMAs such as DAC also involves induction of TEs, triggering an immune response directed against the malignant clone [26–28, 30]. We now demonstrate that this induction of TEs by DAC is enhanced by the addition of ATRA. Of note, the overall more pronounced upregulation of TEs in *TP53*-mutated U937 compared to *TP53* wildtype MOLM-13 is well in line with the more effective repression of TEs by functional p53 compared to mutated p53 species [31]. Still, the partial rather than full overlap of TEs commonly regulated in both cells does not rule out a stochastic regulation of TEs under treatment with DAC + ATRA. In general, TE regulations in human cancer are not fully understood to this day [43]. Notably, TE regulations display considerable inter-patient variation in newly diagnosed, untreated AML patients with distinct disease subtype [44].

As a consequence of TE derepression, induction of RIG-I and the dsRNA sensor MDA5 was observed, supporting the concept of enhanced immunogenicity by unleashing a stronger antileukemic “viral mimicry” response by this drug combination compared to DAC alone. Therefore, studies to prospectively capture alterations in T cell subpopulations during early treatment phases of DAC + ATRA, e.g. by single-cell RNA sequencing, in order to demonstrate the immunomodulatory effects of this treatment, appear warranted. In this regard, the enhanced immunogenicity of AML blasts after derepressive HMA treatment, via presentation of Cancer/testis antigens, TEs and other leukemia-associated antigens, provides a rationale to combine HMAs with ATRA and cellular immunotherapy such as donor-lymphocyte infusions (DLIs) [45] in patients with relapse of AML after allogeneic stem cell transplantation. Hence, the cooperative effects of DAC + ATRA (particularly given the excellent tolerability of ATRA) might also provide clinical benefit in the post-allogeneic transplantation setting.

A major clinical goal is to determine whether the HMA + retinoid approach is also active in *TP53-*mutated AML/MDS, representing—even in young, fit patients—a highly unmet clinical need. These investigations are also driven by the increasing rate of treatment-related AML/MDS, often presenting in older patients and with bi-allelic loss of *TP53* [46]. In the *TP53* mutated cell line U937, cooperative effects of the HMA + retinoid approach are noted. In contrast, cell lines THP1 and HL-60 also harboring *TP53* mutations showed no synergistic antiproliferative effects for DAC + ATRA. In U937 cells the *TP53* mutation is the main oncogenic driver, while THP1 and HL-60 cells also harbor the potent oncogenes *MLL-AF9* and amplified *c-MYC*, respectively. The presence of further strong oncogenic drivers in these cell lines, having undergone adaptation to propagation in vitro for decades, might explain the limited antiproliferative effect observed in THP1 and HL-60 cells compared to U937.

The cooperative effects of the HMA + retinoid in U937 cells are in line with emerging clinical results of a post hoc analysis of the DECIDER cohort, where patients with *TP53* mutations had a higher response rate to DAC + ATRA than patients with wildtype *TP53* [47]. Our model is limited, including only two different cell lines representative of contrary *TP53* status. Identification of a pretherapeutic predictive marker such as RARA expression appears warranted (for the RARA agonist tamibarotene, a higher response rate in AML patients was demonstrated in RARA overexpressors [48]).

In conclusion, we provide in vitro and in vivo evidence for antileukemic cooperativity and epigenetic activity (chromatin remodeling) between DAC and ATRA in non-APL AML. In addition to derepression of tumor suppressor genes such as *HIC1*, induction of transposable elements is observed with both drugs, triggering a “viral mimicry” response. These results warrant further mechanistic studies, e.g., in isogenic AML models displaying different *TP53* states. They are in line with the higher response rate of AML patients with mutated compared to wildtype *TP53* in the DECIDER trial [47]. Given the clinical benefit of ATRA in combination with an HMA, a confirmatory, placebo-controlled randomized phase III trial (DECIDER-2; EudraCT No. 2020-005495-36) is underway. The demonstrated antineoplastic activity of a retinoid in combination with an HMA has implications beyond myeloid malignancies, i.e., also in frequent solid tumor entities, particularly those with a high incidence of *TP53* mutations, such as triple-negative breast cancer and pancreatic cancer.

## Supplementary information

Supplemental Figure 1

Supplemental Figure 2

Supplemental Figure 3 A and B

Supplemental Table 1

Supplemental Table 2

Supplemental Table 3

Supplemental Table 4

Supplemental Table 5

Supplemental Methods

Supplemental data legends

## Data Availability

The datasets generated during and/or analyzed during the current study—if not included in the supplements or accessible via GEO under accession numbers GSE184324, GSE184282, GSE190878 and GSE171053—are available from the corresponding author on reasonable request.
