# Supplementary material for: The antileukemic activity of decitabine upon PML/RARA-negative AML blasts is supported by all-trans retinoic acid: in vitro and in vivo evidence for cooperation
Source: Blood Cancer J. 2022 Aug 22;12(8):122. doi: 10.1038/s41408-022-00715-4 (PMC9395383; doi:10.1038/s41408-022-00715-4)
Supplement: Supplementary file 9 — Supplemental Methods [file 41408_2022_715_MOESM9_ESM.docx]

**Supplemental Methods**

**Cell culture and drug treatment**

Cell lines (U937, MOLM-13, OCI-AML3, MV4-11, THP1, HL-60) were cultured in RPMI 1640 medium (Thermo Fisher Scientific, Waltham, Massachusetts, U.S.A.), supplemented with 100 U/ml penicillin/streptomycin and 10% FCS (Sigma-Aldrich, St. Louis, Missouri, U.S.A.) at 37 °C and 5 % CO_2_. The cells were treated with three daily pulses of 5-aza-2′-deoxycytidine (decitabine, DAC, Selleckchem, Houston, Texas, U.S.A.) dissolved in PBS. One µM all-trans retinoic acid (ATRA, Sigma-Aldrich, St. Louis, Missouri, U.S.A.) dissolved in DMSO was administered after 48 hours. As controls, cells were treated with the appropriate vehicles. Cell numbers and viability were acquired by acridine orange/propridium iodide staining and detection with the Luna ™ fl Dual Fluorescence cell counter (Logos Biosystems Inc., Anyang-si, South Korea). All cell lines were purchased from DSMZ (Leibniz Institute, DSMZ-German Collection of Microorganisms and Cell Cultures GmbH, Braunschweig, Germany) and routinely tested for mycoplasm (all cell lines were mycoplasm-free).

**Caspase-3/7 activity assay**

Caspase 3 and 7 activity was measured with the Caspase-Glo 3/7 Assay System (Promega, Madison, Wisconsin, U.S.A.). A DEVD-bound aminoluciferin substrate and a luciferase were added to harvested cells (U937 and MOLM-13 at 96 and 120 hours). The DEVDase Caspase 3/7 cleaves the DEVD sequence and liberates free aminoluciferin, which is then consumed by the luciferase. This generates a luminescent signal that was detected by the Tecan Spark 10M Luminescence Microplate Reader.

**RNA-sequencing and data analysis**

Read quality was checked with FastQC (≥95 % >Q30), alignment to the reference genome hg38 was performed with STAR, read-counting with htseq-count, differential expression testing with DESeq2 (FDR<0.01; protein-coding transcripts only). GO enrichment analysis was performed with Metascape^12^. For analysis of transposable elements, reads were aligned to modifications provided by the Hammell lab which were condensed compared to the original annotation by filtering out low complexity and simple repeats, rRNA, scRNA, snRNA, srpRNA and tRNA. Read-counting was performed with enabled counting of non-uniquely mapped reads. All analysis steps were calculated on the European Galaxy instance (https://usegalaxy.eu/).

**Assay for Transposase Accessible Chromatin (ATAC)-sequencing and data analysis**

Size selection for 20 to 800 bp fragments and purification of the library was performed twice using Agencourt AMPure XP beads (Beckman Coulter, Brea, California, USA) with a beads-to-sample ratio of 1:2 and 2:3, respectively.  Validation of enriched open chromatin sites was determined by quantitative PCR for constitutively unoccupied TATA-box binding sites (TBP), a specific heterochromatic region on chromosome 18 (chr18) and mitochondrial DNA (mtDNA). Paired-end libraries were sequenced with 40 million reads per sample. Read quality was checked with FastQC (≥95% >Q35), alignment to the GRCh38/hg38 assembly was performed with Bowtie2, PCR duplicates were removed with MarkDuplicates and narrow peaks were called using Genrich (FDR<0.05).

Heatmaps of enriched regions overlapping with POLR2A binding sites and visualization of gene-specific enrichment sites were generated using deepTools2 and HiCExplorer, respectively. These analysis steps were calculated on the European Galaxy instance (https://usegalaxy.eu/). Integrated ChIP-seq tracks for POLR2A and RXRA binding sites originate from ENCODE and from GEO for RARA binding sites (HepG2 cells, GEO accession number GSM2797606).

Differential accessibility analysis was performed using the R package DiffBind v2.14.0 on R v3.6.. In summary, a consensus peak set was defined using peaks from all conditions. Chromatin accessibility at the consensus set of peaks was quantified using the alignments of all samples. As a method of differential analysis edgeR v3.28.0 was applied. To define the treatment effects, group-wise comparisons of each treatment vs control samples were performed*.* Regions with an adjusted *P* value <0.05 and an absolute log2 fold-change >1 were considered as differentially accessible. Annotation of all DARs were performed with the R package ChIPseeker v1.31.3.900 and TxDb.Hsapiens.UCSC.hg38.knownGene v3.10.0. PCA was performed with the base R function *prcomp*. Therefore, log-transformed normalized counts per consensus peaks were generated with the R package DESeq2 v1.26.0 and the function *rlog*.

For the enrichment of transcription factor motifs, the command line tool Homer v4.10 was used on R v3.6.. Therefore, DARs were stratified in opened and closed regions and enriched against a background of consensus peaks. Homer was run with the option *-size given.*

**Quantitative qRT-PCR**

Total RNA was isolated using the RNeasy Kit (Qiagen, Hilden, Germany). First-strand cDNA was generated using random hexamers and the SuperScript Reverse Transcriptase II (Invitrogen, Carlsbad, California, U.S.A.) according to the manufacturer’s protocol. RT-PCR was performed using the LightCycler® 480 SYBR Green I on the LightCycler® 480 Instrument (Roche Life Science) according to the manufacturer’s instructions. The selected target genes were validated using primers listed in Suppl. Table 2. ACTB, RNF20 and TGDS served as reference genes. Normalization was accomplished by Advanced Relative Quantification analysis in the LightCycler® 480 Software. ACTB and RNF20 were purchased as reference gene detection kits via Primerdesign Ltd (Southampton, United Kingdom).

**Analysis of genome-wide DNA methylation data with RnBeads**

Raw intensity data were obtained as IDAT files. DNA methylation data quality control and analysis were performed using the Bioconductor package RnBeads (version 1.10.8). Sites that overlapped with SNPs were filtered, resulting in the removal of 10 131 sites.

Furthermore, probes giving unreliable measurements, 812 as determined by the Greedycut algorithm, were excluded from analysis. As a final outcome of the filtering procedures, 10 943 probes and 0 samples were removed (24 samples and 474 634 probes were retained). The data from the remaining probes were subjected to background subtraction using the methylumi package (method "enmix.oob") and beta-mixture quantile normalization (BMIQ). Hierarchical clustering analysis was performed in R using the Manhattan distance metric and complete linkage criteria.

**Fluorescent Western blot**

Whole cell extracts from cultured cell lines and patient samples were used to perform western blots. LDS sample buffer and Reducing Agent (Invitrogen Novex, Thermo Fisher Scientific, Waltham, Massachusetts, U.S.A.) was added, samples were heated for 5–10 min at 90 °C, loaded onto 4–12% SDS–polyacrylamide gel (Invitrogen Novex, Thermo Fisher Scientific, Waltham, Massachusetts, U.S.A.) and electrophoresed at 180 V for 30 min in MES SDS running buffer (Invitrogen Novex, Thermo Fisher Scientific, Waltham, Massachusetts, U.S.A.). Transfer was performed using a XCell SureLock Electrophoresis Cell at 30 V for 90 min using PVDF membrane (Merck Millipore, Burlington, Massachusetts, U.S.A.) and transfer buffer (Invitrogen Novex, Thermo Fisher Scientific, Waltham, Massachusetts, U.S.A.; 20% methanol, 1% antioxidant). The membrane was blocked in blocking buffer (5 % bovine serum albumin (BSA) in TBS-T (Tris-buffered saline with 0,1 % Tween-20, pH 7.6.) for 60 min, shaking at room temperature. The membrane was incubated overnight rocking at 4 °C. The next day, the membrane was washed three times for 10 min in TBS-T. Multiplexed IRDye secondary antibodies (LI-COR) were used at a dilution of 1:20 000 in 5 % BSA TBS-T, and the membrane was incubated for 60 min at room temperature. After washing with TBS-T, the signals were detected using the 700 nm and 800 nm channels of the Odyssey CLx imaging system operated by the Image Studio software (LI-COR). A list of antibodies used in this study is provided in Suppl. Table 1.
