# Supplementary material for: The antileukemic activity of decitabine upon PML/RARA-negative AML blasts is supported by all-trans retinoic acid: in vitro and in vivo evidence for cooperation
Source: Blood Cancer J. 2022 Aug 22;12(8):122. doi: 10.1038/s41408-022-00715-4 (PMC9395383; doi:10.1038/s41408-022-00715-4)
Supplement: Supplementary file 10 — Supplemental data legends [file 41408_2022_715_MOESM10_ESM.docx]

**Suppl. Fig. 1: Commonly up- or downregulated genes by DAC+ATRA are involved in leukocyte differentiation, immune response and transcriptional initiation.**

Addition to Fig. 2 D. Top 20 results for the GO analysis of genes commonly regulated by DAC+ATRA in U937 and MOLM-13.

**Suppl. Fig. 2: Differential accessibility analysis.** Differential accessibility analysis comparing ATRA vs. untreated (CNTL; middle panel), DAC vs. CNTL (left panel), and DAC+ATRA vs. CNTL (right panel). Pie charts (top row) and volcano plots (bottom row) indicate number of differentially accessible regions (DARs), defined by an adjusted (adj.) p-value <0.05 and an absolute log2 fold change >1. Red dots, increased accessibility in ATRA, DAC, or DAC +ATRA-treated samples; blue dots, increased accessibility in CNTL samples. This comparison of chromatin accessibility at the union set of all peaks revealed major effects of the different drug treatments on the overall chromatin compaction. Specifically, differential accessibility analysis revealed 17,784 regions with a significant decrease (adj. p-value <0.05 and log2 fold change <-1) and 16,606 regions with a significant increase (adj. p-value <0.05 and log2 fold change >1) in accessibility in ATRA- compared to CNTL samples. As expected, DAC treated samples showed an overall increase in chromatin accessibility with 30,940 regions with increased accessibility. This effect was further enhanced in the double-treated samples, with 31,460 regions with increased accessibility in DAC+ATRA vs. untreated.

**Suppl. Fig. 3: DAC and ATRA cooperate in inducing “viral mimicry”.** (A) U937 cells (technical triplicates) untreated and treated with DMSO, DAC, ATRA and DAC+ATRA were analyzed for mRNA expression of *ERV3-1* and *ZNF117* at 72 and 120 h. RT-qPCR was performed as described in Supplemental Methods, for primers see Suppl. Table 2. (B) Overview of the anti-viral immune signaling pathway of RIG-I-like receptors.

**Suppl. Table 1: List of antibodies.** Antibodies used for Western Blot.

**Suppl. Table 2: Primers for qPCR.** Primers used for RT-qPCR in AML cell lines.

**Suppl. Table 3: Patients’ characteristics.** Characteristics of 15 AML patients treated with

DAC or DAC+ATRA.

**Suppl. Table 4: Patients expression array 1.** 9 genes exhibiting induction by DAC alone, and significantly higher induction by DAC+ATRA.

**Suppl. Table 5: Patients expression array 2.** 60 genes in order of the strongest difference (Diff) between induction by DAC alone and downregulation by DAC+ATRA.
